# How is loneliness related to anxiety and depression: A population‐based network analysis in the early lockdown period

**DOI:** 10.1002/ijop.12851

**Published:** 2022-05-06

**Authors:** Marcin Owczarek, Emma Nolan, Mark Shevlin, Sarah Butter, Thanos Karatzias, Orla McBride, Jamie Murphy, Frederique Vallieres, Richard Bentall, Anton Martinez, Philip Hyland

**Affiliations:** ^1^ Faculty of Life and Health Sciences Ulster University Coleraine UK; ^2^ Department of Psychology University of Sheffield Sheffield UK; ^3^ School of Health and Social Care Edinburgh Napier University Edinburgh UK; ^4^ Trinity Centre for Global Health Trinity College Dublin Dublin Ireland; ^5^ Department of Psychology Maynooth University Maynooth Ireland

**Keywords:** Anxiety, Depression, Loneliness

## Abstract

High risk of mental health problems is associated with loneliness resulting from social distancing measures and “lockdowns” that have been imposed globally due to the COVID‐19 pandemic. This study explores the interconnectedness of loneliness, anxiety and depression on a symptom level using network analysis. A representative sample of participants (*N* = 1041), who were of at least 18 years of age, was recruited from the Republic of Ireland (ROI). Loneliness, anxiety and depression were assessed using validated instruments. Network analysis was used to identify the network structure of loneliness, anxiety and depression. Loneliness was found to be largely isolated from anxiety and depression nodes in the network. Anxiety and depression were largely interconnected. “Trouble relaxing,” “feeling bad about oneself” and “not being able to stop or control worrying” were suggested as the most influential nodes of the network. Despite the expectation that loneliness would be implicated more robustly in the anxiety and depression network of symptoms, the results suggest loneliness as a distinct construct that is not interwoven with anxiety and depression.

In response to the escalating COVID‐19 pandemic governments around the globe placed extensive restrictions on the movement of people and imposed “lockdowns” which forced their populations to remain in their homes. There were concerns that the lockdown would increase loneliness and exacerbate mental health problems (Rossi et al., 2020). Furthermore, the World Health Organisation (WHO) has expressed its concerns over how the measures of self‐isolation and quarantine may lead to an increase in loneliness, anxiety, depression and suicidality (WHO, NaN).

The current global pandemic has seen cities placed under quarantine and entire populations being forced to restrict their movements, for all but essential purposes. It is possible that this may result in decreased social connectedness and increased loneliness. Theories of loneliness suggest that connectedness, both social and physical, are essential human needs (Cacioppo & Patrick, 2008). The extant literature suggests a complex and interdependent relationship between social isolation, loneliness, and adverse psychological health outcomes with researchers suggesting that loneliness is caused by perceived social isolation (Twenge et al., 2003). Studies have shown that loneliness is a predictor of depression and anxiety and recently Santini et al. (2020) demonstrated that social isolation increased feelings of perceived isolation, which in turn predicted higher depression and anxiety symptoms. However, this study also demonstrated that a bi‐directional effect was present, whereby higher depression and anxiety symptoms predicted higher amounts of perceived isolation and loneliness. Research therefore suggests that loneliness is connected to anxiety and depression scores. However, it is not yet known which direction this relationship may occur. These finding suggest that loneliness, depression and anxiety symptoms are interrelated; connected by an intricate interplay between symptoms that may manifest as an intertwining of symptoms, underpinned by multiple and possibly cascading pathways of development (Afzali et al., 2017). In recent studies, examining the mental health of the UK population during the COVID‐19 pandemic at the initial stages of lockdown, preliminary evidence suggests that clinically relevant levels of generalised anxiety and depression (16–28%) are common (Shevlin et al., 2020). Also, studies examining GAD and depression in the ROI found that 27.7% screened positive for GAD or depression during the first week of the strict COVID‐19 lockdown measures (Hyland et al., 2021).

A review of the psychological impact of quarantine, due to COVID‐19, revealed numerous negative emotional outcomes, including, increased stress, depression, anxiety and stigma associated with quarantine measures (Santini et al., 2020). Loneliness and social isolation frequently co‐occur—the term “loneliness” often refers to subjective evaluation of one's experience while “social isolation” is often used in the literature as referring to the extent and frequency of social interactions (Hwang et al., 2020). Previous studies suggest that social isolation incurred by lockdowns can influence loneliness, anxiety and depression (Hyland et al., 2021). Therefore, given the lockdown and other social restriction measures implemented during the current global pandemic, examining the symptom‐level connectedness of these covariates is a useful approach to explore their symptom manifestation. This has the potential to shed light on how the symptoms feed into each other.

Governments and policy makers need take into account the benefits and risks of lockdown for public health. Understanding the impact that strict social distancing rules have on a population, in respect to mental health, will aid in assessing the use of this strategy if a future out‐break occurs. In addition, it is an opportunity to understand how symptoms of anxiety, depression and loneliness are related (Hyland et al., 2021). The present study used self‐reported (subjective) measures of loneliness, anxiety and depression, during the first wave and the beginning of the national lockdown. It is therefore provides a base level for comparison as the lockdown continued and the COVID‐19 environment changed. With continuing restrictions and isolation measures in place, the question of how loneliness is conceptually and empirically distinct from other disorders such as anxiety and depression is warranted.

The purpose of this study was to use a network analysis to examine the degree to which loneliness is associated with anxiety and depression symptoms in the general population. Given the date of this data collection was at the beginning of government enforced lockdown, this study will provide baseline estimates of how loneliness anxiety and depression are related. In addition, based on the possible connections between these disorder symptoms, this study aimed to discover if there are “bridge symptoms,” or central connections between disorders (anxiety, depression, loneliness). Therefore, there are three goals of this study. First, to investigate the degree of relatedness between loneliness, anxiety and depression in a nationally representative sample obtained shortly after initial distancing measures were introduced. Particular interest will be placed upon bridge symptoms to examine the connectedness of symptoms across symptom clusters. The second goal was to identify the central variables in this network by inspecting network node centrality measures to determine which variables influence the network the most. Third, is to examine symptom clusters which may potentially guide future interventions. Taken together, the results of our study may contribute to our growing understanding of the symptom manifestations of loneliness, anxiety and depression.

## METHODS

### Recruitment and sample

Ethical approval for the study was granted by the Sheffield University ethical review board. Participants (*N* = 1041) were recruited by the survey company Qualtrics, using an online research panel that was representative of the adult population of the Republic of Ireland (ROI). Data collection commenced on the 31st March 2020, 31 days after the first confirmed Covid‐19 case in the ROI and just 19 days after initial distancing measures were implemented. Data gathering process concluded on the 5th of April 2020.

Stratified quota sampling (in accordance with Irish census from 2016) was employed to ensure representativeness of the sample. Participants had to be 18 years or older when the survey took place and able to complete the survey in English. Participants were requested to participate by the survey company (Qualtrics) via e‐mail. Consenting participants cmpleted the survey online and were reimbursed for their time. Women constituted 51.5% of the sample. The age of the participants ranged from 18 to 88 with a mean age of 44.97 years (*SD* = 15.76). Sociodemographic characteristics are reported in Murphy et al. (2021). There were no missing values present in the data.

Ethical approval for the study was granted by the ethical review board of the University of Sheffield and Ulster University. The data used was gathered as a part of the Longitudinal COVID‐19 Psychological Research Consortium (C19PRC) study and was funded with support from the University of Sheffield, Ulster University, and the University of Liverpool which was secured during the COVID‐19 pandemic in March 2020 to support the collection of data for the first two waves of the C19PRC study. UKRI/ESRC funding for this study was obtained in May 2020 (Grant ref.: ES/V004379/1). The authors declare they have no conflict of interest. Informed consent was obtained from all individual adult participants included in the study.

### Measures

#### Anxiety

Symptoms of anxiety were measured using the Generalised Anxiety Disorder 7‐item Scale (GAD‐7: Spitzer et al., 2006). The participants were presented with seven questions relating to symptoms they have experienced over the last 2 weeks. The items were to be endorsed on a 4‐point likert scale (0—“Not at all,” 3—“Nearly every day”) and the scores range between 0 and 21. The GAD‐7 has been shown to produce scores of high reliability and validity in community studies (Spitzer et al., 2006), and the reliability of the scores in the current sample was high (α = .94).

#### Depression

Symptoms of depression were measured using the Patient Health Questionnaire 9 (PHQ‐9; Kroenke et al., 2001). The participants were presented with nine questions relating to symptoms of depression that they have experienced during the past 2 weeks. The items were presented on a 0–3 likert scale (0—“Not at all,” 3—“Nearly every day”) and the scores ranged from 0 to 27. The PHQ‐9 is a widely used questionnaire of high validity and reliability (Kroenke et al., 2001). In the present sample, the reliability of the scores was high (α = .90).

*Loneliness*: Loneliness was measured using the Three‐Item Loneliness Scale (Hughes et al., 2004). Participants were asked how often do they “lack companionship,” “feel lonely” and “feel left out.” The scale has been previously utilised which contributes to its validity (Mullen et al., 2019). The possible answers were (a) “Hardly ever,” (b) “Some of the time” and (3) “ften.” Scores ranged from 3 to 9. Reliability of the scores in the current sample was high (α = .87).

### Statistical analysis

First, the associations between loneliness, anxiety and depression were assessed at the construct level by correlating the total scale scores. Second, the item‐level network analysis was conducted using a Gaussian Graphical Model (GGM) using maximum likelihood (ML) estimation and the “ggmModSelect” model search option with stepwise estimation (Epskamp et al., 2018). Using this allows for the estimation of unregularized models which are based on 100 glasso estimations that are then re‐estimated without regularisation. The stepwise portion of this iteratively adds and removes edges until an optimal BIC is obtained. This was performed both for network estimation and, later, the bootstrap analyses. In the case of the present study, network nodes represent quantitatively measured symptoms of anxiety, depression and loneliness. Edges are interpreted as lines whose thickness is based on partial correlation coefficients between the nodes. The network was visualised using the “spring” layout which places strongly connected nodes closer together (Epskamp et al., 2018). The network included nine nodes representing PHQ‐9 items, seven nodes representing the GAD‐7 items and three nodes representing the items from Three‐Item Loneliness Scale.

Centrality was assessed; nodes of a network differ in their importance. High centrality nodes act as strongly connected “hubs” linking together more peripheral nodes. Low centrality nodes are characterised by being on the margins of the network and/or having fewer and weaker connections to other nodes. To highlight symptoms which are the most influential within the network, three commonly used centrality indices were calculated. *Betweenness* indicates the number of shortest paths connecting any two symptoms (Opsahl et al., 2010). *Closeness* indicates how easily, when starting from a specified node, the flow of information reaches all the other nodes. High closeness indicated the likelihood that a node would be affected by changes in other nodes within the specified network (Opsahl et al., 2010). As the network included negative correlations between nodes, the *Expected Influence* (EI) was chosen as it was previously suggested to perform better than the commonly used “Strength” index when negative correlations are present (see Robinaugh et al., 2016). EI aims to assess the influence a node holds over its immediate neighbours.

Bootstrapped difference tests were performed using the R package “bootnet” to determine network reliability (Epskamp et al., 2018). The procedure uses observed difference in edge values and bootstrapping to determine the 95% confidence intervals (CIs). When the 95% CI crosses zero, it is suggestive of the edges being not statistically different. Case‐dropping subset bootstrap method was used to determine the stability of the centrality indices. This method re‐estimated the network using increasingly smaller representative samples from the original sample and estimated correlations between the newly‐generated and original indices—smaller decrease in correlation is indicative of higher stability (Epskamp et al., 2018). Bootstrapping procedures were conducted using 1000 iterations.

Then, to establish empirically derived communities of symptoms, Clique Percolation Method was utilised (Lange, 2019). This method is useful for psychometric analysis as it allows for nodes to belong to more than one community while also allowing (other) nodes to not belong to any community. This method detects groups of nodes that are fully connected (called *k*‐cliques) that are defined as adjacent under the condition of sharing all but one node—adjacent cliques are then grouped into communities. Using a permutation test, two crucial parameters for clique percolation can be established: *k*—determining the size of cliques and the intensity (*I*)—a numerical value indicating how strongly the cliques have to be connected to be considered a community. The analysis allowed *k* to vary between 3 and 6 and the *I* to vary between 0.40 and 0.01—allowing for a wide range of possible solutions.

To estimate which edges are most important to the network in terms of connecting different constructs (loneliness, depression, anxiety), bridge expected influence (BEI; Jones et al., 2019) was calculated using the “*networktool*” R package. BEI identifies “bridge” nodes by examining only the cross‐construct EI of a node. This estimation allows for establishing which nodes are the key contributors to the co‐occurrence of diagnostic clusters.

## RESULTS

Descriptive statistics and correlations for the total scale scores for the main study variables are shown in Table 1. All scales scores were highly positively correlated.

**TABLE 1:** Descriptive statistics and correlation for main study variables

	Loneliness total	PHQ total	GAD total
Loneliness total	1.00		
PHQ total	.560^**^	1.00	
GAD total	.530^**^	.805^**^	1.00
Mean	4.97	5.79	5.03
*SD*	1.867	6.096	5.521
Min–max	3–9	0–27	0–21
Range	6	27	21

*Note*: **Correlation is significant at the .01 level (2‐tailed).

**Figure 1:**
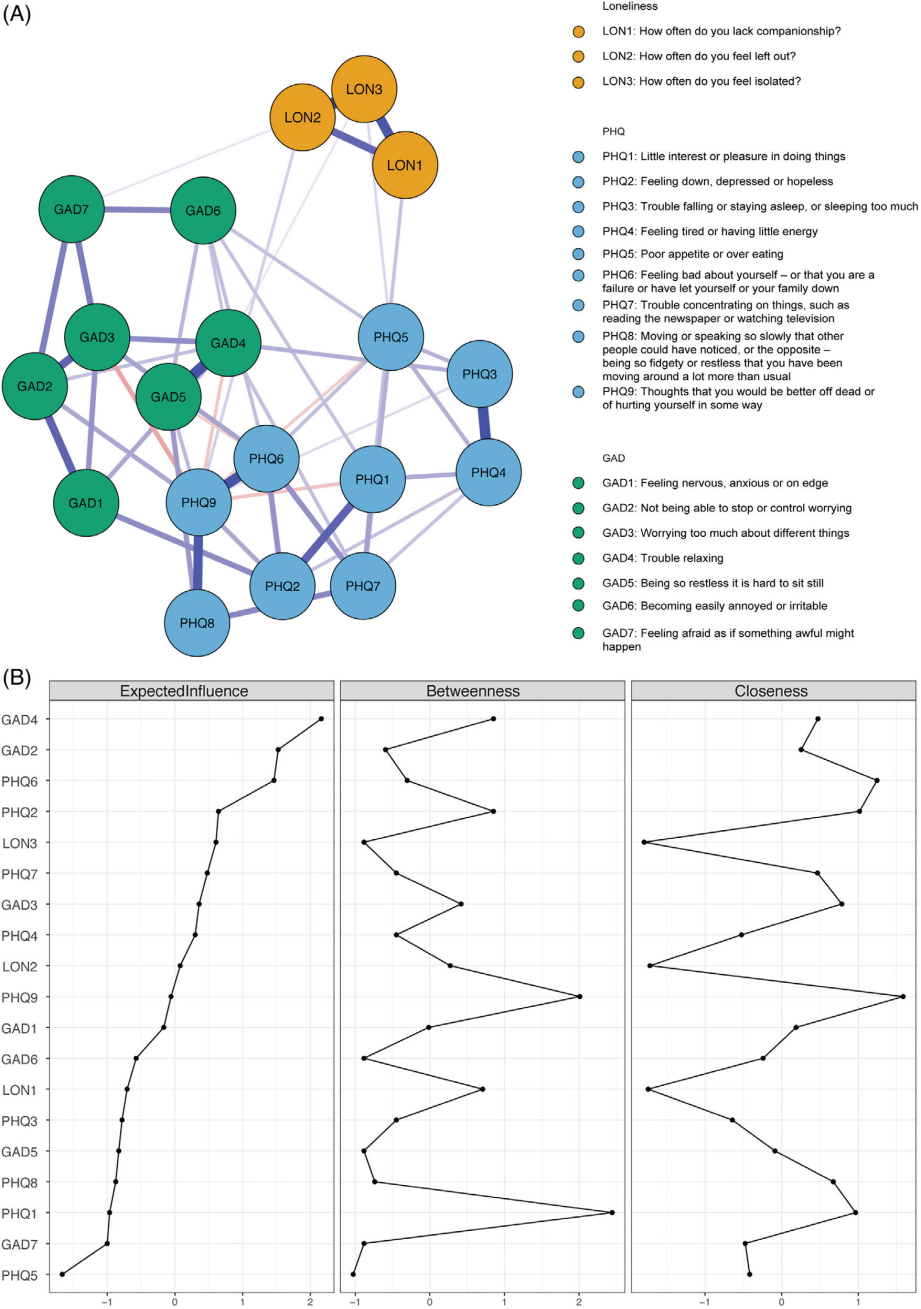
(a) Visualised network. Note: Blue edges represent positive associations and red edges represent negative associations between nodes. (b) Standardised Centrality measures. Note: Values for centrality are standardised and sorted from least to most Expected Influence.

Out of the possible 171 edges, 52 (30.41%) were estimated as being different from zero using the “*ggmModSelect*” estimation. The weights matrix is shown in Table 2 and the network is presented in Figure 1a.

**TABLE 2:** Edge weights matrix for loneliness, depression and anxiety items

	Loneliness			PHQ									GAD						
	1	2	3	1	2	3	4	5	6	7	8	9	1	2	3	4	5	6	7
Ln1	0.00																		
Ln2	0.31	0.00																	
Ln3	0.41	0.51	0.00																
PHQ 1	0.12	0.00	0.00	0.00															
PHQ 2	0.00	0.00	0.00	0.33	0.00														
PHQ 3	0.00	0.00	0.00	0.00	0.00	0.00													
PHQ 4	0.00	0.00	0.00	0.17	0.12	0.44	0.00												
PHQ 5	0.00	0.00	0.08	0.00	0.00	0.15	0.15	0.00											
PHQ 6	0.00	0.00	0.00	0.00	0.21	0.00	0.00	0.13	0.00										
PHQ 7	0.00	0.00	0.00	0.19	0.00	0.00	0.12	0.16	0.21	0.00									
PHQ 8	0.00	0.00	0.00	0.00	0.00	0.00	0.00	0.00	0.00	0.23	0.00								
PHQ 9	0.00	0.09	0.00	−0.12	0.17	0.07	0.00	−0.10	0.43	0.00	0.39	0.00							
GAD 1	0.00	0.00	0.00	0.00	0.22	0.00	0.00	0.00	0.00	0.00	0.00	0.00	0.00						
GAD 2	0.00	0.00	0.00	0.00	0.00	0.00	0.00	0.00	0.00	0.00	0.00	0.17	0.33	0.00					
GAD 3	0.00	0.00	0.00	0.00	0.00	0.00	0.00	0.00	0.18	0.00	0.00	−0.18	0.21	0.33	0.00				
GAD 4	0.00	0.00	0.05	0.00	0.00	0.16	0.00	0.00	0.00	0.13	0.00	−0.11	0.17	0.13	0.22	0.00			
GAD 5	0.00	0.00	0.00	0.00	0.00	0.00	0.00	0.00	−0.10	0.00	0.20	0.13	0.00	0.00	0.00	0.45	0.00		
GAD 6	0.00	0.00	0.00	0.12	0.00	0.00	0.00	0.12	0.13	0.00	0.00	0.00	0.00	0.00	0.00	0.11	0.14	0.00	
GAD 7	0.00	0.06	0.00	0.00	0.00	0.00	0.00	0.00	0.00	0.00	0.00	0.00	0.00	0.25	0.25	0.00	0.00	0.24	0.00

When inspecting betweenness, closeness and expected influence of the network (Figure 1b), loneliness items were among the items of medium Expected Influence and Betweenness. The Loneliness nodes also represented the lowest values of the Closeness centrality measures. These are probably due to the items representing loneliness being tightly clustered and largely isolated from the rest of the network—exerting high influence on other loneliness items but not anxiety and depression (Figure 1a).

GAD4 (“Having trouble relaxing”) held the highest EI (2.17). In addition, the item presented high betweenness. Among the depression items, PHQ6 (“Feeling bad about yourself—or that you are a failure or have let yourself or your family down?') presented the highest EI (1.46), with low Betweenness. Both of these anxiety and depression items also presented high closeness values, while GAD6 (“Becoming easily annoyed or irritable”) and PHQ5 (“Poor appetite or overeating”) presented the lowest values (GAD6 betweenness: −0.88 and PHQ5 betweenness: −1.02).

**Figure 2:**
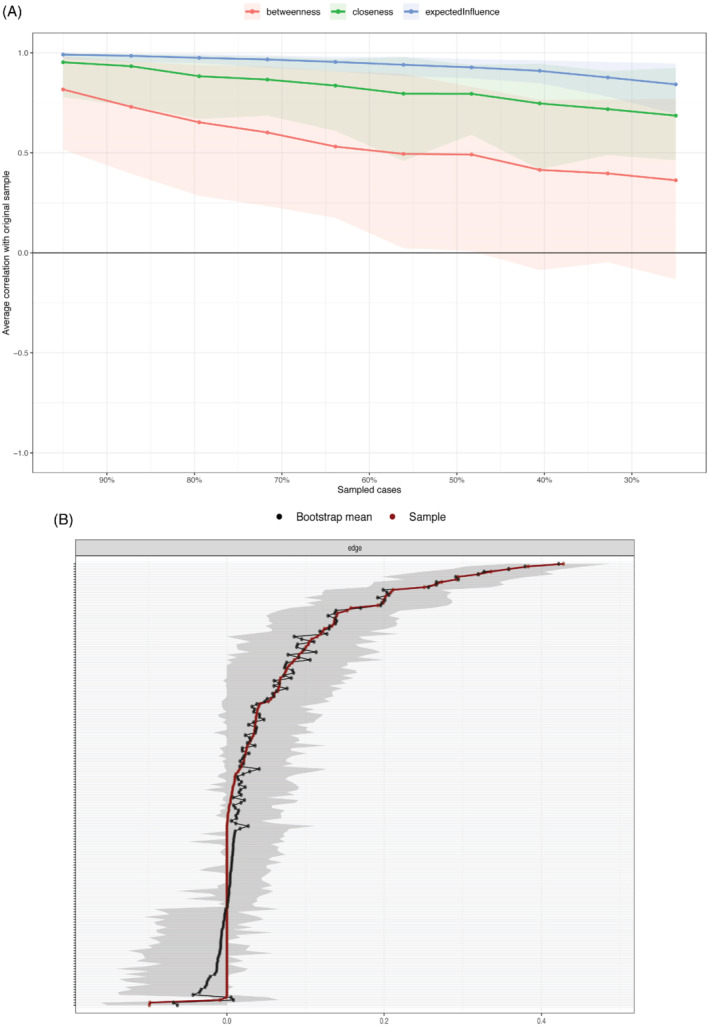
(a) Case‐drop stability analysis. Note: Mean correlations between centrality values of original sample and bootstrapped sub samples with different degrees of persons dropped. Lines reflect means and areas around the lines reflect 95% CIs. (b) Network Stability. Note: The red line represents the edge, as estimated in the sample. The grey indicates 95% bootstrapped confidence interval. The *x*‐axis represents the edges, while specific edges are denoted along the *y*‐axis by the grey lines.

**Figure 3:**
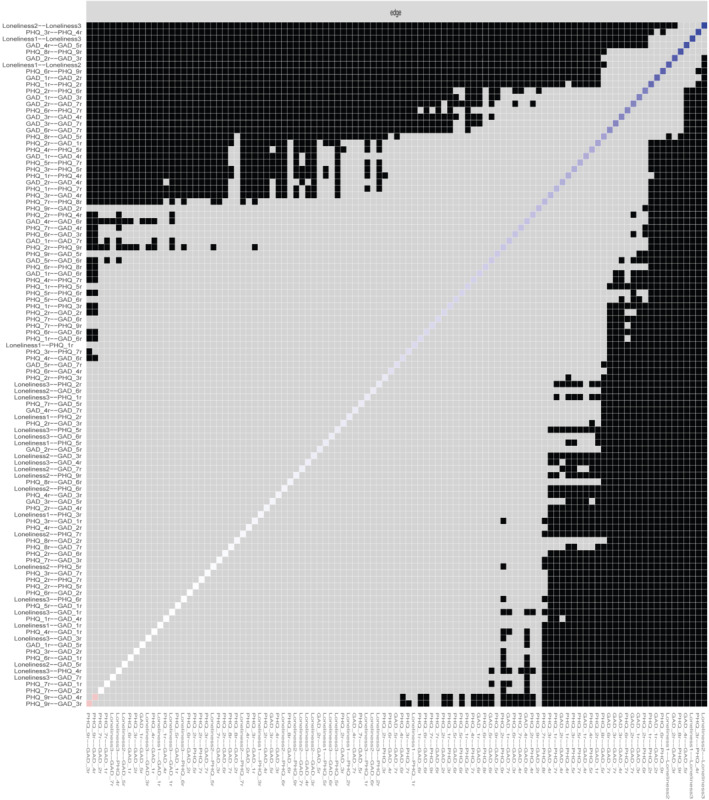
Edge stability. A black box on the intersection of a row and a column indicates a significant difference (*p* < .05) while a grey box indicates no significant difference.

The bootstrapping procedure supports robustness of two of the centrality measures used and edge weights (based on the bootstrapped confidence intervals of the edge weights—Figure 2a). The bootstrapped case‐dropping stability analyses of the network suggested that both Expected Influence and Closeness centrality indices showed good stability (both coefficients were above .5). Betweenness stability coefficient was low (.01) indicating that the index should be interpreted with a degree of caution. The results of the case‐dropped correlation stability analysis is presented in Figure 2b. Furthermore, the bootstrapped difference test (Figure 3) indicated that the rank ordering of edge weights (i.e., thickness of edges) could be interpreted with confidence.

**Figure 4:**
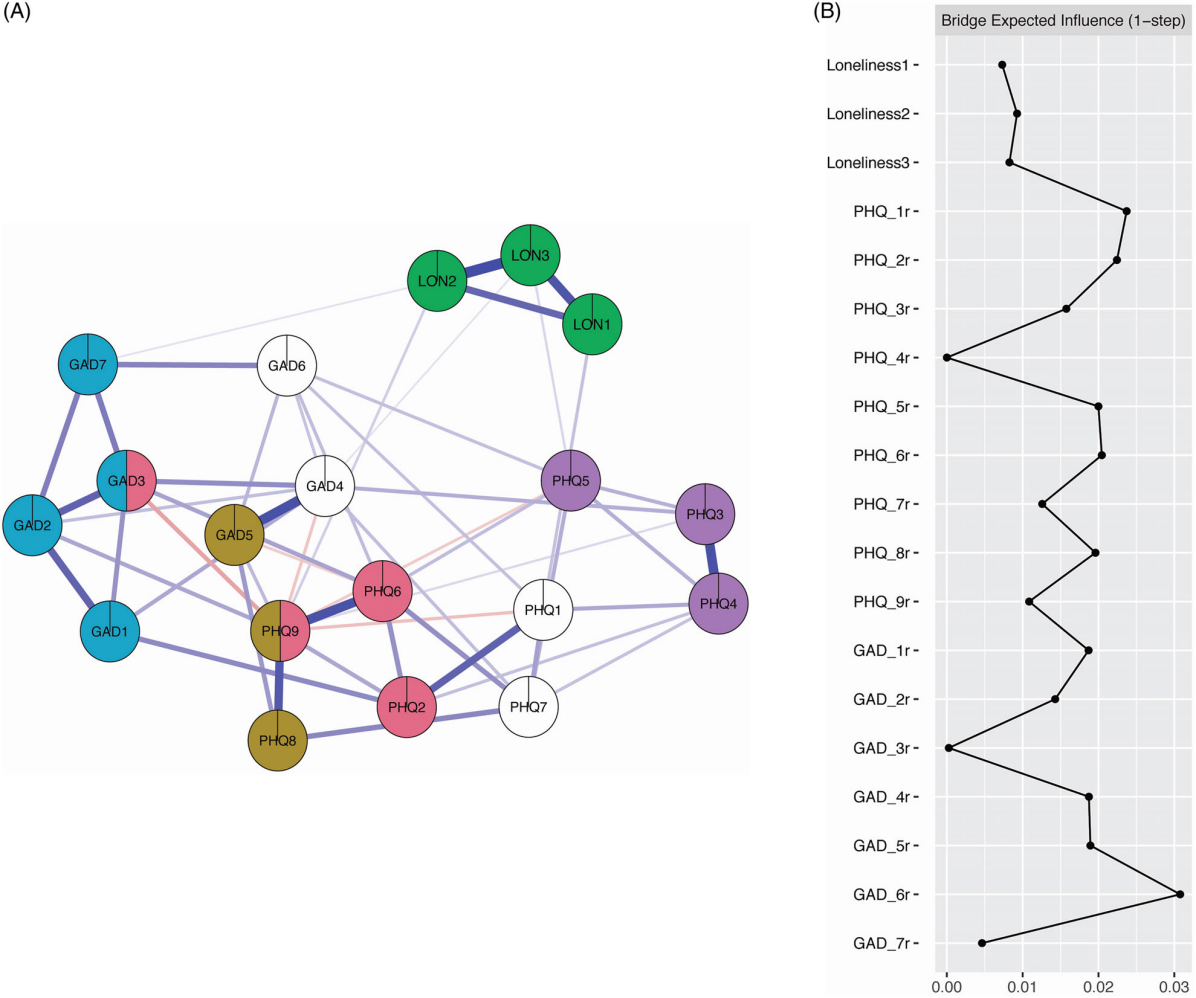
(a) Clique Percolation. Note: Colours reflect the Clique percolation community analysis results with white nodes indicating a node does not belong to any community. (b) Bridge expected influence. Note: Values for Bridge Expected Influence are standardised.

The results of Clique Percolation are presented in Figure 4a. The analysis suggested that *k* value of 3 with *I* = 0.225 was the best solution. A total of five communities were detected with PHQ1 (“Little interest or pleasure in doing things”), PHQ7 (“Trouble concentrating on things, such as reading the newspaper or watching television”), GAD4 (“Trouble relaxing”) GAD6 (“Becoming easily annoyed or irritable”) not belonging to any community. Community 1 (presented as pink in Figure 4a) included depression items of low mood, worry, suicidality/self‐harm and low‐self esteem with one anxiety item representing excessive worry. Community 2 (dark yellow) included depression items representing psychomotor retardation, suicidality/self‐harm and a single anxiety items representing restlessness. Community 3 (green) included items of Loneliness and did not include any nodes from other constructs. Community 4 (blue) included only anxiety items that represented feeling nervous/anxious/on‐edge, uncontrollable worry, excessive worry and fear. Community 5 (violet) included three depression items which represented sleep problems, having low energy and a change in appetite for food.

The standardised estimates of bridge expected influence (1‐step) are presented in Figure 4b. Items GAD6 (“Becoming easily annoyed or irritable”) and PHQ1 (“Little interest or pleasure in doing things”) showed highest values (BEI 0.03 and 0.02, respectively). Items from the loneliness cluster showed low BEI. This, along with the results from the Clique Percolation, suggests that anxiety and depression clusters are better connected to each other than they are to the loneliness cluster.

## DISCUSSION

This study used a network approach to examine loneliness, anxiety and depression. The first purpose of this study was to examine how self‐reported feelings of loneliness influenced the manifestation of anxiety and depression symptoms in the general population assessed during COVID‐19‐related social distancing restrictions. Node centrality, communities and cluster bridges of the network were examined.

The network exhibited high stability both in terms of structure but also two of the three centrality measures (Expected Influence and Closeness). Betweenness indices have been previously observed to display wide confidence intervals (Bringmann et al., 2019) and such is the case in this study. Therefore, in further interpretation of the node centrality, the focus will be placed on EI and Closeness centrality measures as these were sufficiently stable. The results of this examination enable the identification of which symptoms are potential targets of future clinical interventions. Nodes exhibiting high EI in a network were previously suggested to be prime targets for such interventions (e.g.,, see Robinaugh et al., 2016; Boschloo et al., 2016). Our findings suggested that, on a symptom level, GAD4 (“Trouble relaxing”) and PHQ6 (“Feeling bad about yourself…”) are two of the most influential nodes. However, due to the present examination being cross‐sectional, it is not possible to determine whether most influential nodes are in fact responsible for causality of other symptoms or are the causal end points in the network. In addition, the Closeness centrality measure, which can be interpreted as describing the rate to which a change within the node is going to affect other nodes and how quickly the node itself will change based on the changes in other nodes, was the lowest among the loneliness items (Opsahl et al., 2010). This suggests that intervening on the disorders (anxiety and depression) themselves is theoretically going to bring about a more rapid change within the network.

The results of this study found that loneliness existed as a distinct construct that is more distant to anxiety and depression than the two disorders are to each other. This has been supported by the results from the Clique Percolation, by examining closeness centrality and by examining bridge centrality. Studies have demonstrated that bridge symptoms are implicated in the emergence of comorbidity structures between mental disorders (Cramer et al., 2010). Targeting central and bridge symptoms might constitute a focal point of therapies, as they are suggested to accelerate the development of network interactions between symptoms (Borsboom, 2017). Jones et al. (2019) found that deactivating bridging symptoms was more effective for preventing symptom activation, suggesting that bridging symptoms are implicated in the cascade of symptom activation. Bridge centrality for loneliness was low, there was no indication of significant “bridge symptoms” from loneliness to anxiety or depression in the network analysis—meaning symptoms of loneliness did not have strong direct connections to other neighbouring symptoms or clusters of anxiety and depression. This suggests that loneliness symptoms do not share any common symptoms with anxiety or depression, nor affect these disorders strongly, thus is a distinct disorder (Borsboom, 2017). Further examining bridge EI suggests that GAD6 (“Becoming easily annoyed or irritable”) and PHQ2 (“Feeling down depressed or hopeless”) were highly influential. Given that, one has to consider that the psychological interventions are not “surgical” tools being able to affect only one of the symptoms in a network. Rather, these interventions are performed using verbal communication or visual and auditory stimuli and as such, and in the foreseeable future, affecting only one psychological symptom is unrealistic (Eronen, 2020). However, the present study can be used as a guide when considering the impact and trajectory of these changes. If an intervention was to be planned—this study suggests that loneliness, anxiety and depression while distinct, are interconnected phenomena that affects change and development of the other. Furthermore, there are some reports of utilising network analysis in predicting future onset of psychological ailments (Boschloo et al., 2016) and as such, there exists initial support for examining highly central symptoms in the light of having high prognostic impact on the risk of developing a disorder. As such, while longitudinal research is needed, the present study supports loneliness being a worse predictor of anxiety and depression than the other two constructs are to each other.

Interestingly, the Clique Percolation results suggest that two of the communities (Figure 4a) include symptoms both constructs of anxiety and depression. This supports the growing literature that put's the distinction of these disorders into question (Gomez et al., 2020; Kaiser et al., 2021). Furthermore, this finding reinforces the notion that anxiety and depression are more strongly interwoven when compared to loneliness which exhibited no cross‐construct clustering. These results, as well as the results of the overall network partially align with previous findings. For example, Kaiser et al. (2021) have also identified communities that span across anxiety and depression in a psychiatric sample. However, when compared to Kaiser et al. (2021) and Beard et al. (2016), the most central symptoms differed albeit slightly—both of the studies suggest PHQ2 (“Feeling down, depressed or hopeless”) GAD2 (“Not being able to stop or control worrying”) and GAD3 (“Worrying too much about different things”) as exerting the highest influence (or strength in the case of Beard et al.) while the present study suggests only GAD2 as being the most influential anxiety symptom with PHQ 6 (“Feeling bad about yourself—or that you are a failure or have let yourself or your family down”) as the most influential depression symptom which was only moderately influential in the mentioned studies. These differences might stem from the present study utilising a nationally representative sample as opposed to clinical samples.

The results from the present examination do not support the notion that addressing loneliness is an effective way of diminishing anxiety and depression. The role loneliness plays as an effective positive influencer of both anxiety and depression while, from a network analysis perspective, not being interwoven with these symptoms needs to be addressed. These seemingly contradicting results could suggest that there exist constructs that act as bridges between loneliness and the other two constructs that have not been captured by the present study. Alternatively, future research could approach loneliness, anxiety and depression as being governed by a higher order latent construct (e.g., purposelessness). Identifying these could give rise to new, effective targets for intervention under the conditions where being isolated is mandatory both during a pandemic (e.g., Lockdown) and beyond (e.g., social anxiety, care‐homes, hospices, etc.). Previous research into the effects loneliness has on an individual's sense of meaning might provide possible venues of examination. Stillman et al. suggest that “Meaning itself is acquired socially. Hence to be cut off from others is potentially to raise the threat of losing access to all socially mediated meanings, purposes, and values.” (Stillman et al., 2009). In addition, social isolation was found to increase self‐defeating behaviour, aggression, dilated time perception, meaninglessness, lethargy, lack of emotion and a decrease in self‐awareness (Twenge et al., 2003).

This study had some limitations. Aside from being based on a representative sample, the data used in this study was cross‐sectional, therefore, causal effects between the symptoms could not be established. Another limitation of this study is the relatively low mean anxiety and depression scores. There is preliminary evidence that symptom network connectivity differs between clinical and nonclinical populations, as this was a general population sample, these results may not extend to a clinical sample. The data used within this study was collected during early days of “lockdown,” however, the straightforward extent of these measures and adherence to them was not controlled for in this study. This presents a confound as these might vary across participants and influence other variables used in the analysis. The loneliness measure used is also of particular interest when considering the limitations it incurs. Namely, the measure uses only three items to measure the extent of one's loneliness, it is a subjective measure and therefore other confounding factors (i.e., subjectivity of the participant) may influence how one perceives their level of loneliness. An interesting line of research that could remedy this issue would be to compare objective (isolation—e.g.,, the number of social interactions) and subjective (loneliness) measures. Furthermore, there exists no conclusive evidence that the translation of central symptoms obtained from network analysis is a straightforward endeavour. Only limited evidence exists that network analysis can inform therapists as to what symptoms are needed to be targeted in interventions (Rodebaugh et al., 2018). The study also did not account for socioeconomic, anthropogenic and demographic factors which may have influenced the results. An interesting line of future research could include examination of the symptom manifestation of anxiety depression and loneliness while taking these factors into account.

In conclusion, despite the expectation that loneliness would be implicated more robustly in the anxiety and depression network of symptoms, overall, the results suggest loneliness as a distinct construct, with no indication of meaningful “bridge symptoms” from loneliness to anxiety or depression. What this could mean for public health interventions is that, under conditions where population levels of loneliness are increased, for example, as an effect of the government imposed “lockdown,” reducing the severity of other symptoms is a more viable strategy of influencing the symptom network of anxiety and depression.
